# Somatic mutations of KIT in familial testicular germ cell tumours

**DOI:** 10.1038/sj.bjc.6601880

**Published:** 2004-05-18

**Authors:** E A Rapley, S Hockley, W Warren, L Johnson, R Huddart, G Crockford, D Forman, M G Leahy, D T Oliver, K Tucker, M Friedlander, K-A Phillips, D Hogg, M A S Jewett, R Lohynska, G Daugaard, S Richard, A Heidenreich, L Geczi, I Bodrogi, E Olah, W J Ormiston, P A Daly, L H J Looijenga, P Guilford, N Aass, S D Fosså, K Heimdal, S A Tjulandin, L Liubchenko, H Stoll, W Weber, L Einhorn, B L Weber, M McMaster, M H Greene, D T Bishop, D Easton, M R Stratton

**Affiliations:** 1 Section of Cancer Genetics, Institute of Cancer Research, Brookes Lawley Building, 15 Cotswold Road, Sutton, Surrey SM2 5NG, UK; 2 Genetic Epidemiology Division, Cancer Research UK Clinical Centre, St. James's University Hospital, Leeds LS9 7TF, UK; 3 Cancer Research UK Genetic Epidemiology Unit, Strangeways Research Laboratory Worts Causeway, Cambridge CB1 8RN, UK; 4 Department of Medical Oncology, Division of Medicine, University of New South Wales and Prince of Wales Hospital Randwick, Sydney, Australia; 5 Department of Haematology and Medical Oncology Peter MacCallum Cancer Centre, St Andrews Place, East Melbourne, Victoria 3002, Australia; 6 Princess Margaret Hospital and University of Toronto, 610 University Avenue, Toronto, ON, Canada M5G 2M9; 7 Department of Radiotherapy and Oncology, University Hospital, V Uvalu 84, 150 06 Prague, Czech Republic; 8 Department of Oncology 5073, Rigshospitalet, Copenhagen, Denmark; 9 Génétique Oncologique EPHE Faculté de Médecine Paris-Sud, UPRESS 1602 and Service d'Urologie, CHU, 94276 Le Kremlin-Bicêtre, France; 10 Department of Urological Oncology, Phillips University, Marburg, Germany; 11 Department of Chemotherapy C and Department of Molecular Genetics National Institute of Oncology, Rath Gyorgy u. 7, H-1122 Budapest, Hungary; 12 Department of Medical Oncology, St James Hospital, Dublin, Ireland; 13 Pathology/Laboratory For Experimental Patho-Oncology, Erasmus University Medical Center Rotterdam/Daniel den Hoed Cancer Center, Josephine Nefkens Institute, 3000 DR Rotterdam, Netherlands; 14 Cancer Genetics Laboratory, University of Otago, PO Box 56, Dunedin, New Zealand; 15 Department of Oncology, The Norwegian Radium Hospital and Department of Medical Genetics, Rikshospitalet 0027 Oslo, Norway; 16 Laboratory of Clinical Genetics, Institute of Clinical Oncology, NN Blokhin Russian Cancer Research Center, Kashirskoye sh., 24, Moscow 115478, Russian Federation; 17 Clinical Cancer Research Unit, UICC Familial Cancer and Prevention Project Heuberg 16 CH-4051 Basel, Switzerland; 18 Indiana University, Indianapolis, IN, USA; 19 Abramson Family Cancer Research Institute, University of Pennsylvania, PA USA; 20 Clinical Genetics Branch, Division of Cancer Epidemiology & Genetics , National Cancer Institute National Institutes of Health , 6120 Executive Boulevard, Room Rockville, MD 20852- 7231, USA

**Keywords:** KIT, testicular germ cell tumours

## Abstract

Somatic mutations of the KIT gene have been reported in mast cell diseases and gastrointestinal stromal tumours. Recently, they have also been found in mediastinal and testicular germ cell tumours (TGCTs), particularly in cases with bilateral disease. We screened the KIT coding sequence (except exon 1) for germline mutations in 240 pedigrees with two or more cases of TGCT. No germline mutations were found. Exons 10, 11 and 17 of KIT were examined for somatic mutations in 123 TGCT from 93 multiple-case testicular cancer families. Five somatic mutations were identified; four were missense amino-acid substitutions in exon 17 and one was a 12 bp in-frame deletion in exon 11. Two of seven TGCT from cases with bilateral disease carried KIT mutations compared with three out of 116 unilateral cases (*P*=0.026). The results indicate that somatic KIT mutations are implicated in the development of a minority of familial as well as sporadic TGCT. They also lend support to the hypothesis that KIT mutations primarily take place during embryogenesis such that primordial germ cells with KIT mutations are distributed to both testes.

Testicular germ cell tumours (TGCTs) are the most common malignancy in males between the age 15 and 45 years (Ferlay *et al*, 2001). There are several risk factors for TGCT including previously diagnosed TGCT, undescended testis (UDT) and a family history of the disease. TGCT has been one of the highest familial relative risks of any cancer syndrome with reported increased risks of 8–10-fold to brothers and 4–6-fold to fathers (Forman *et al*, 1992; Heimdal *et al*, 1996). We previously described linkage of familial testicular cancer to a locus (*TGCT1*) at Xq27 (Rapley *et al*, 2000). This locus was particularly strongly associated with families characterised by at least one case of bilateral testicular cancer. The results indicated, however, that only a minority of families are attributable to this locus and that additional TGCT susceptibility genes are likely to exist.

The KIT gene encodes a type III transmembrane tyrosine kinase receptor. KIT is expressed in several cell types where it regulates primordial germ cell migration, proliferation and apoptosis during foetal gonad development (Mauduit *et al*, 1999). KIT has been shown to be expressed in some TGCT (Strohmeyer *et al*, 1995; Bokemeyer *et al*, 1996) and somatic mutations in KIT have recently been identified in testicular (Tian *et al*, 1999) and mediastinal germ cell tumours (Przygodzki *et al*, 2002). Mutations have been reported in a high proportion of patients with bilateral disease, and in a much smaller proportion of unilateral cases (Looijenga *et al*, 2003). When both tumours from bilateral cases could be examined, the same mutation was present in both tumours. Together, these results suggest that somatic KIT mutations occur early in embryogenesis, before the primordial germ cells have divided and migrated to the gonads. As a consequence, primordial germ cells with KIT mutations are distributed to both testes and hence KIT mutations are associated with bilateral disease (Looijenga *et al*, 2003).

Previous studies have indicated that KIT mutations found in germ cell tumours are somatic. To investigate further the role of KIT in predisposition to TGCT, and the role of somatic mutations in familial tumours, we have examined a series of constitutional and tumour DNAs from patients with TGCTs and a family history of the disease.

## MATERIALS AND METHODS

The International Testicular Cancer Linkage Consortium (ITCLC) has obtained samples from 326 families with two or more cases of TGCT (Table 1

**Table 1:** Pedigree structure of cases with family history of TGCT used in KIT mutation search

**Family type**	**Pedigrees (by type) identified by ITCLC**	**Number of pedigrees analysed (by type) using constitutional DNA**	**Number of pedigrees analysed (by type) using DNA from tumour material**
Sib trios	8	5	2
Large⩾3 affected cases	31	22	3
Sib pairs	154	106	43 (11)^a^
Father/son pairs	52	46	16 (2)^a^
Cousin pairs	40	33	14
Uncle/nephew pairs	32	23	13 (2)^a^
Grandfather/grandson pairs	4	5	1
Monozygous twins	4	0	0
Great grandfather/great grandson pairs	1	0	1
			
Total	326	240	93

a Number in brackets represents pedigrees (by subtype) for which only tumour material was available for analysis, these families are not included in the KIT analysis of constitutional DNA samples.

). For this study, we analysed DNA extracted from blood lymphocytes (constitutional DNA) from one affected individual from each of 240 families, for whom the DNA was most readily available. The pedigree structure for these families is shown in Table 1. We also examined tumour materials from 123 cases from 93 families; for 15 of these families, the tumour material only was available and these were therefore not included in the 240 constitutional DNA set (Table 1). Seven tumours were from patients with bilateral disease, but the tumour material was only available from one of each pair of tumours arising in these patients.

Patients donated samples and medical information with full informed consent and with local or national ethical review board approval. Information on clinical status including type of TGCT, age of diagnosis, presence of UDT and laterality of disease was confirmed by reviewing histological reports and clinical notes.

DNA was prepared from whole blood and from formalin-fixed, paraffin-embedded tumour sections using standard techniques. The tumour material was microdissected to minimise contamination by surrounding normal tissue. Primer sequences for KIT were designed from the KIT mRNA and genomic sequence (Ensembl gene ID=ENSG00000157404), using the Primer3 software (http://www-genome.wi.mit.edu/c gi-bin/primer/primer3_www.cgi). Primer sequences for the 21 KIT exons and PCR product sizes are shown in Table 2

**Table 2:** KIT gene primer pairs

**Exon**	**Forward**	**Reverse**	**PCR product size**
Exon2a	AATAGCAGGGCAGCTTTGTC	GTTGGTGCACGTGTATTTGC	358
Exon2b	CTTGGCAGGCTCTTCTCAAC	CCTTCTAGACCCAGCCAGAA	395
Exon3a	GTGCGTGATACATGGAAAGC	GTAGGCGCGTTTCACACTTT	397
Exon3b	GCTTCTATAGATCCTGCCAAGC	AGGTGGATCAACGAGAAGAGA	372
Exon4	GATAGGTTAGCACCATGCTTTG	TCTCCCAGACAATCCACCTC	400
Exon5	TGGAGAAGTTAATTGCTGCTATTTT	TCATTCATTCAGTGATAACAAAATTC	389
Exon6	GGAAATCAACCAATTGTTTTTG	TCGTGGATTTACGGGTTACA	384
Exon7	CCTCAAACAGGCATAGATTTCC	AACCACCAAACCACGAAGTC	364
Exon8	TTCTGCCCTTTGAACTTGCT	AAAGCCACATGGCTAGAAAAA	386
Exon9	ATGCCACATCCCAAGTGTTT	TGACATGGTCAATGTTGGAA	364
Exon10	AACCAAGGTGAAGCTCTGAGAC	CTCCTCAACAACCTTCCACTG	384
Exon 10 small	ATCCCATCCTGCCAAAGTT	CTGTGGGGAGAAAGGGAAA	246
Exon11	TAGCTGGCATGATGTGCATT	GGCGCAATTTCACAGAAAAC	397
Exon 11 small	AGAGTGCTCTAATGACTGAGACAA	AAACAAAGGAAGCCACTGGA	279
Exon12	ATTGCGCCCCTTTTGATAG	GTTCAGACATGAGGGCTTCC	392
Exon13	TGCTCAAGCGTAAGTTCCTG	GCAAGAGAGAACAACAGTCTGG	335
Exon14a	CTCCACATAAGGCTGCTTTT	CCCATGAACTGCCTGTCAAC	381
Exon14b	TCTCACCTTCTTTCTAACCTTTTC	TCAGCAAAAATCTAGGTTTGAATC	390
Exon15	TGTAGCAAAGGGGATGAGGA	CCCTAACTGCCATTGACCAT	335
Exon16	GATCTGCCTGCAAGTTCACA	AAAACACAAAACTCTTTAGAGAATCAC	385
Exon17	CATCATTCAAGGCGTACTTTTG	TCCCTAGACAGGATTTACATTATGA	399
Exon 17 small	TAAATGGTTTTCTTTTCTCCTCCA	TTCGATAAAATTGTTTCCTGTGA	233
Exon18	CTCCACATTTCAGCAACAGC	GGCTGCTTCCTGAGACACA	333
Exon19	AAGTGGATGGCACCTGAAAG	CCCTCAACATCTGGGTTTCT	390
Exon20	TCCATATGTCCAGTTGCATAGC	GCCCAATTTGCAACCTAAGA	350
Exon21	TTCCATCAGTTAGTTGTGATCTTG	GACAAAAATCATCGGCCACT	390

. Exons>400 bp were amplified using overlapping primer pairs. A total of 23 primers pairs were used to examine the coding region of the KIT gene. Primers designed for exon 1 failed to amplify under a variety of PCR conditions and were redesigned but again failed to give a PCR product. Exon 1 was therefore not examined. Mutations of KIT are predominantly located in exons 10, 11 and 17 (Pignon *et al*, 1997; Tian *et al*, 1999; Rubin *et al*, 2001; Przygodzki *et al*, 2002; Looijenga *et al*, 2003); therefore, the tumour material was examined only at these exons. Primers generating a smaller sized PCR fragment were designed for exons 10, 11 and 17 to allow easy amplification from paraffin-embedded material and were specifically used to analyse the tumour material.

For constitutional DNA, all exons were examined by conformation sensitive gel electrophoresis (CSGE) (Ganguly *et al*, 1993). Briefly, both PCR primers were labelled with adenosine 5′[*γ*-^32^P]triphosphate by T4 polynucleotide kinase. After amplification, PCR products were heated to 98°C and cooled down to 60°C over 30 min to allow heteroduplex formation. PCR samples were run on a CSGE gel (10% v v^−1^ ethanediol, 13.75% v v^−1^ formamide, 15% v v^−1^ acrylamide with 4 mg ml^−1^ piperazine and 1 × GTB buffer (89 mM Tris, 29 mM taurine and 0.5 mM EDTA). Polymerase chain reaction (PCR) products from samples that showed migration shifts on CSGE were bidirectionally sequenced using the BigDye terminator v3 sequencing kit and a 3100 automated sequencer (Applied Biosystems, Warrington, UK).

All tumour samples were examined for exons 10, 11 and 17 of the KIT gene by direct sequencing. The tumour material was amplified and sequenced using specifically designed primers that generated a smaller sized PCR fragment than those designed for CSGE analysis and allowed for ease of amplification from tumour material. Sequencing was performed using the BigDye terminator v3 sequencing kit and a 3100 automated sequencer (Applied Biosystems).

Differences in distribution between categorical variables were assessed with the appropriate contingency table test.

## RESULTS AND DISCUSSION

In total, 240 constitutional DNA samples from TGCT cases with a positive family history were examined using CSGE. Two conservative nonsynonymous constitutional sequence variants were detected, M541L and V399I (Table 3

**Table 3:** Sequence variants in KIT detected in constitutional DNA from patients with familial TGCTs

**Exon**	**Nucleotide change RefSeq NM_000222**	**Amino-acid change**	**Number of cases**
3	G525A	A168A	1
6	C999T	N326N	1
7	G1216A	V399I	1
10	A1642C	M541L	43
10	A1659G	K546K	5
16	C2370T	L783L	1
17	C2451T	I798I	12
18	G2607C	L862L	53
19	G2643A	P874P	1

). M541L was found in 43 out of 240 (17.9%) cases and it is a common polymorphism found in 32 out of 192 (16.6%) of normal controls. V399L was found in only a single case and was not found in 200 controls. V399 is not conserved in other species (mouse, zebrafish and xenopus) and the amino-acid substitution is conservative, suggesting that this variant is a rare polymorphism rather than a disease-causing change. Overall, the results provide no evidence that germline KIT mutations are associated with an increased risk of testicular cancer.

Somatic mutations of KIT were detected in five out of 123 TGCTs examined (Table 4

**Table 4:** Sequence changes in KIT detected in TGCTs

**Sample name**	**Exon**	**Nucleotide change RefSeq NM_000222**	**Amino-acid change**	**Case details giving tumour type, age at diagnosis, history of undescended testis and family history**
2158-201	11	Del 1675–1686	del MYEV AA 552–555	R. sem^a^ and L.ITGCN
				Age at diagnosis=39 years
				History of bilateral UDT
				Family history=sib pair
274-201	17	A2480G	D820G	L. sem
				Age at diagnosis=52 years
				Family history=MZ twins (twin brother has bilateral disease, no tumour available for this patient)
295-304	17	G2467C	D816H	R. mixed and L. sem^a^
				Age at diagnosis=39 and 55 years
				History of UDT
				Family history=sib trio
377-1664	17	G2467T	D816Y	R NS
				Age at diagnosis=66 years
				Family history=father/son pair
285-201	17	G2467T	D816Y	L. sem
				Age at diagnosis=39 years
				Family history=sib pair

a Tumour examined in bilateral cases. Sem=Seminoma; NS=nonseminoma; ITGCN=intratubular germ cell neoplasia; R=right side; L=left side; UDT=undescended testis.

and Figure 1

**Figure 1:**
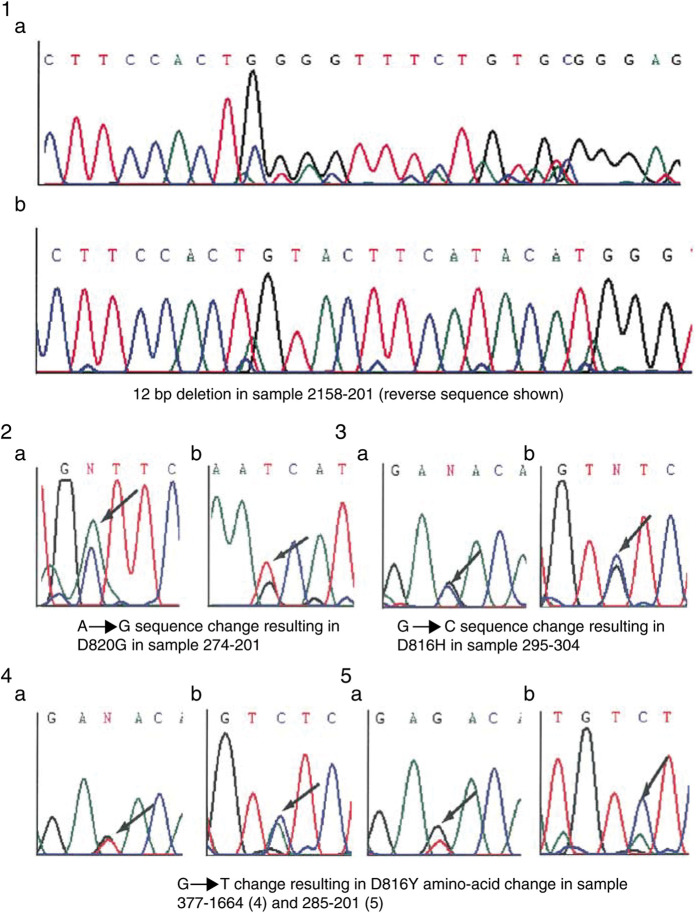
Chromatograms showing sequence variants in KIT gene sequence in testicular tumours: (1a) exon 11 reverse sequence showing 2158-201 12 bp deletion; (1b) exon 11 wild-type sequence; (2) tumour 274-201 forward (a) and reverse (b) exon 17 sequence showing A → G variant; (3) tumour 295-304 forward (a) and reverse (b) exon 17 sequence showing G → C change; (4) tumour 377-1664 forward (a) and reverse (b) exon 17 sequence showing G → T variant; (5) tumour 285-201 forward (a) and reverse (b) exon 17 sequence showing G → T variant.

). Three mutations involved codon 816, a known hotspot for KIT mutations in testicular (Tian *et al*, 1999; Przygodzki *et al*, 2002; Looijenga *et al*, 2003) and other cancers (Rubin *et al*, 2001). Another mutation involved codon 820, an infrequently mutated residue but one that has been previously reported (Pignon *et al*, 1997). The fifth mutation was a 12 bp deletion encoding a 4 amino-acid in-frame deletion in the cytoplasmic juxamembrane domain of KIT. In-frame deletions of this region are common in gastrointestinal stromal tumours (GIST) (Rubin *et al*, 2001) but have not been documented in TGCT. All these mutations were shown to be somatic. Two out of seven (28.5%) familial bilateral cases carried a somatic KIT mutation compared with three out of 116 (2.6%) familial unilateral cases (*P*=0.026, Fisher's exact test). Unfortunately, samples of the other tumour/ITGCN from the two bilateral cases with KIT mutations were not available to evaluate the presence of the mutations. While the frequency of KIT mutations in unilateral TGCT is similar to that detected previously, the proportion of cases with bilateral disease is much lower despite the fact that we examined a larger proportion of the KIT gene than in the study by Looijenga *et al* (2003). The reason for this is unclear. However, it may indicate that bilateral disease in the context of familial testicular cancer has a different pathogenesis from sporadic bilateral cases and that most of the familial bilateral cases are explained by the elevated risk conferred by the underlying susceptibility genes. Nevertheless, the overall pattern of an elevated frequency of KIT mutations in bilateral compared to unilateral cases supports the observation of Looijenga *et al* (2003) and suggests that somatic KIT mutations may take place early in development.

In conclusion, our results indicate that constitutional mutations of KIT are not associated with a substantially increased risk of TGCT. Somatic mutations of KIT are found in familial TGCT tumours with a higher proportion in cases with bilateral disease. Overall, the proportion of KIT mutations in TGCT is low and other somatic and susceptibility genes must play important roles.
